# The Impact of Adding Chitosan Nanoparticles on Biofilm Formation, Cytotoxicity, and Certain Physical and Mechanical Aspects of Directly Printed Orthodontic Clear Aligners

**DOI:** 10.3390/nano13192649

**Published:** 2023-09-26

**Authors:** Botan Barzan Taher, Tara Ali Rasheed

**Affiliations:** 1 Department of Pedodontics, Orthodontics and Preventive Dentistry, College of Dentistry, University of Sulaimani, Sulaymaniyah 46001, Iraq; tara.rasheed@univsul.edu.iq; 2 College of Dentistry, American University of Iraq-Sulaimani, Sulaymaniyah 46001, Iraq

**Keywords:** chitosan nanoparticles, 3D printed aligner, antibiofilm, cytotoxicity, accuracy, degree of conversion, deflection force and tensile strength

## Abstract

Aligner treatment is associated with bacterial colonization, leading to enamel demineralization. Chitosan nanoparticles have been demonstrated to have antibacterial properties. This in vitro study aims to determine the effect of adding chitosan nanoparticles to directly 3D-printed clear aligner resin with regard to antibiofilm activity, cytotoxicity, degree of conversion, accuracy, deflection force, and tensile strength. Different concentrations (2%, 3%, and 5% *w*/*w*) of chitosan nanoparticles were mixed with the clear resin, and the samples were then 3D printed. Additionally, the thermoforming technique for aligner manufacturing was utilized. The obtained specimens were evaluated for antibiofilm activity against *Streptococcus mutans* bacteria and cytotoxicity against L929 and 3T3 cell lines. Additionally, Fourier transform infrared spectroscopy via attenuated total reflection analysis was used to assess the degree of conversion. Geomagic Control X software was utilized to analyze the accuracy. In addition, the deflection force and tensile strength were evaluated. The results indicated a notable reduction in bacterial colonies when the resin was incorporated with 3 and 5% chitosan nanoparticles. No significant changes in the cytotoxicity or accuracy were detected. In conclusion, integrating biocompatible chitosan nanoparticles into the resin can add an antibiofilm element to an aligner without compromising the material’s certain biological, mechanical, and physical qualities at specific concentrations.

## 1. Introduction

Digital tools have been involved in all aspects of healthcare, including dentistry; orthodontics has done so in every patient’s diagnosis, treatment, data-recording appliance design, and fabrication. Clear aligners, made from precise impressions or digital scans of a patient’s teeth, gradually align teeth by applying digitally planned calibrated forces. The preference for aligner treatment and the increase in its prevalence can be attributed to patient demand for “invisible” treatments and the limitations of conventional fixed orthodontics [1]. Additionally, it is more aesthetically pleasing, more comfortable, and easy to use, providing a more hygienic replacement for fixed orthodontic treatment, enhancing periodontal health, and possibly shortening clinical duration [2].

The aligner therapy has a less frequent rate of white spot lesions (WSLs) and exhibits a broader surface area of WSLs versus fixed mechanotherapy [3]. However, the process is lengthy, labor-intensive, and costly.

The direct 3D printing technique for aligner manufacturing allows for digital design and creates transparent aligners, which are considered to be the next fundamental shift [4]. Compared to thermoforming aligners, 3D-printed aligners pose comparable or better mechanical properties [5]. Homogeneity and intra-aligner thickness customization are achievable with 3D printed aligners (which may reduce the crucial need for attachments); this possibility of adding heterogenicity in the thickness within the 3D printed aligner alters the magnitude of the exerted forces [6]. It also enhances accuracy and fitness [7], particularly on undercuts, which can be improved. Furthermore, the aligner edges are smooth and require neither trimming nor polishing, resulting in cost-effectiveness and less laboratory work [8].

Nonetheless, the 3D printing technique reveals limitations that may pose future solutions for seldom available software, high purchasing requirements, and restricted material availability [9]. Additionally, there is a potential concern regarding the toxicity of uncured resin used in 3D printing [10]. Furthermore, biofilm deposition on the tooth structure may induce dental caries. Therefore, it is vital to create preventative approaches to assist orthodontic patients in managing biofilm and improving oral health [11]. 

Numerous efforts have attempted to involve antimicrobial characteristics in orthodontic appliances and auxiliary materials, and research on implementing nano-antibacterial materials in orthodontics is also expanding. However, nanomaterials have not yet attained the ideal balance between antimicrobial efficacy and biocompatibility. Chitosan nanoparticles (Chs) have a wide range of applications and have long been employed with or without other substances and techniques to increase their functionality [12,13]. Adding Chitosan, a minimally toxic agent with antibacterial properties and the ability to reduce enamel demineralization, to 3D-printed aligner resin materials may decrease the incidence of WSLs [14,15,16]. Currently, there is limited research on including nanoparticles in aligners’ material for microbe inhibition [17], and, to the best of our knowledge, none of the research has assessed and compared the effect of adding Chs to an aligner’s resin. Thus, this study assessed the effect of Chs incorporated into the aligner’s resin on biofilm formation by *Streptococcus mutans* bacteria, categorized as one of the most virulent bacteria to cause enamel lesions. 

Furthermore, cytotoxicity testing of the mixture (CR + Chs) is considered a fundamental step in evaluating the biocompatibility of dental materials because it minimizes the need for in vivo or human tests [18]. Before polymerization, 3D printed materials are toxic and gradually become less toxic at the end. Post-curing and processing are critical for achieving the recommended toxicity levels for 3D printing materials [10]. Moreover, unreacted or unaffected double bonds and the number of residual monomers in terms of the degree of conversion of the experimental combinations were assessed at the end of curing as a marker for the mixture’s formulation, temporal stability, clinical behavior, biocompatibility, and mechanical features [19,20]. 

Additionally, the nanoparticles’ inclusion might alter the accuracy and mechanical properties of the produced aligners. Typically, an aligner’s mechanism of action involves a precisely designed and manufactured aligner that exerts substantial controlled forces to move each tooth into the desired position without deforming or tearing the material. The force is generated from the mismatch between the aligner material and the tooth structure [21], which can be reflected by the deflection force and the tensile strength of the aligner material, which the present study measures. 

## 2. Materials and Methods

### 2.1. Fabrication of the Specimens

The Ethical Committee of the College of Dentistry, University of Sulaimani, approved this investigation. This research was designed as an in vitro experimental study. The Chs powder (low molecular weight of deacetylated chitin, Sigma Aldrich, code: 448869-50G, Steinheim, Germany) was incorporated in clear resin (CR) (Dental LT Clear resin V2, Formlabs, Millbury, OH, USA) and dispersed using an ultrasonic processor (UP100H, Hielscher Ultrasonics, Teltow, Germany) at 100 W, 30 kHz for 5 min under standard room temperature and storage times for all samples without light exposure [22,23]. Moreover, the weight percentages of Chs powder to CR were 2%, 3%, and 5% using an electric balance (Kern aes 200-4C, sensitivity: 0.0001 gm, KERN & Sohn GmbH, Balingen, Germany). The composition of the CR is illustrated in Table 1 [8].

The design and STL (stereolithography) file of models were manufactured in different shapes and sizes according to each test via Blender (version 3.2.2, Blender Foundation, Amsterdam, The Netherlands) and Autodesk (version 3.5.474, Autodesk, San Francisco, CA, USA), except for the accuracy test, for which an STL file from the scanned maxillary model (Nissin dental model, Nissin, Japan) was utilized by CEREC Primescan (Sirona Dental Systems, Bensheim, Germany). Then, the STL files of each test were exported to pre-printing software (Preform, version 3.24.1, Formlabs, Somerville, MA, USA) to customize and prepare the samples for 3D printing, later to be printed by a 3D printer (Form 3B, Formlabs, Somerville, MA, USA). The layer thickness (100 µm) was set up in the 3D printer according to manufacturer instructions. After 3D printing, the samples were washed by Form wash (Formlabs, Somerville, MA, USA) for 10 min with triphenylene glycol monomethyl ether to remove the unpolymerized resin. The samples then underwent post-curing (Form cure, Formlabs, Somerville, MA, USA) for 60 min under 60 °C, according to manufacturer instructions. The supports were removed and polished with 150-, 220-, 400-, 800-, 1000-, and 2000-grit wet sandpapers with water [24]. 

Thermoforming sheet material (Thr) (Zendura, Circle, dimensions; 125 × 1.02 mm, Bay Materials, Fremont, CA, USA) was prepared as a control, according to manufacturer instructions (code: 172; temperature: 220 °C; heating time: 55 s; and pressure: ≥ 5 bar) for the thermoforming machine (Biostar, Scheu-Dental, Iserlohn, Germany) [25]. The Thr sheet was molded over a polished round stainless steel disk (thickness = 10 mm, diameter = 70 mm). Later, the samples for each test were cut and removed from the flat portions of the thermoformed foils in accordance with their intended shapes.

The dimensions of all samples were measured at the center of the samples using a digital micrometer gauge and then disinfected for five minutes with 70% ethanol, followed by phosphate-buffered saline (PBS). Each sample was placed in a sealed sterilizing pouch and exposed to UV radiation for 60 min in a biosafety cabinet. 

### 2.2. Antibiofilm Test

The antibacterial efficiency of the material against the biofilm formation of *Streptococcus mutans* (ATCC-25175, LYFO Disk, Microbiologics, Grenoble, France) on the surface of specimens was measured after 24 h. For this purpose, five groups of ten sterilized specimens (disks) were prepared (diameter = 5 mm and thickness = 1 mm) (n = 10): Thr; CR; 2% Chs incorporated into CR (CR + 2% Chs); 3% Chs incorporated into CR (CR + 3% Chs); and 5% Chs incorporated into CR (CR + 5% Chs).

The bacterial pellet was activated by immersing it in 2 mL of nutrient broth and incubating it for 2 h at 37 °C in a sterile tube in anaerobic conditions. Then, the bacteria suspension (100 µL) was dropped into the nutrient broth (10 mL) and incubated for 24 h. Thereafter, 1 mL of bacterial solution was added to the nutrient broth (9 mL) along with the sample, which was then incubated for 24 h. The extracted bacteria’s turbidity was justified to be 0.5 McFarland (1.5 × 10^8^ colony-forming units per milliliter (CFU/mL)) by utilizing a spectrophotometer (Lambda 25, UV/VIS Spectrophotometer, PerkinElmer, Shelton CT, USA) to measure the absorbance (abs.) value at a wavelength of 600 nm. Next, each sterilized disc specimen was extracted from the bacterial suspension and rinsed twice with PBS (pH 7.2) to eliminate nonadherent cells. After this, the specimen was placed in a tube containing a sterile saline solution (10 mL), and the adhered bacteria cells were dispersed via a vortex for 10 min. Later, a serial dilution was carried out until (10^3^), after which the dilution solution was pipetted out (0.1 mL) onto the surface of the nutrient agar plate. After this, the plates were incubated at body temperature (37 °C, 24 h) under anaerobic conditions. The test was repeated three times to confirm the reliability of the anti-biofilm effect of the experimented groups. As in earlier investigations, a direct cell culture approach determined the number of CFU/mL of grown bacteria [26,27].

### 2.3. Cytotoxicity Assay

There were ten samples per group: G1, Thr; G2, CR; G3, CR + 2% Chs; G4, CR + 3% Chs; and G5, CR + 5% Chs. Similar dimensions to the former tests were used. Two negative control groups, distal water (DW) and polyethylene (PE), and a positive control group, ethanol 70% (ET), were added to the cytotoxicity assay [28].

#### 2.3.1. Cell Preparation and Proliferation

The L929 cells (ATCC: CCL-1) as mouse fibroblasts and 3T3-L1 (ATCC: CL-173) as mouse embryonic fibroblast cell lines were acquired from the National Center of Genetic Resources in Iran and were used in this experiment. The cells were grown in Dulbecco’s Modified Eagle Medium (DMEM) media with 10% fetal bovine serum (FBS) (Gibco, Life Technologies, Thermo Fisher, Waltham, MA, USA) and incubated in a cell incubator until the desired density was reached. The liquid extract sample ratio was determined by the ratio of the surface area or mass of the test sample to the volume of extractant used (3 cm^2^/mL), as the thickness of the sample is > 0.5 mm, as recommended by ISO parameters [29]. Every three days, the medium was changed. Furthermore, antibiotics (10,000 IU/mL penicillin and 10,000 µg/mL streptomycin) were added to the cell culture.

#### 2.3.2. Transfer of Cells

The below procedure was used to perform cell passaging and culturing in a bigger vessel for cell proliferation. First, the cells’ previous media were removed, and all remaining media was washed out of the cells using 1 mL of phosphate-buffered saline (PBS) buffer. After this, each flask was filled with 1 mL of trypsin-EDTA (Sigma Aldrich, Taufkirchen, Germany) solution (0.25% trypsin and 1 mM EDTA), which was then incubated for three to five minutes to cause the cells to separate from the culture vessel. A 15 mL falcon tube containing the separated cells was filled with trypsin solution. The function of trypsin was inhibited by adding 2 mL of culture medium containing 10% FBS and centrifuging it at 300× *g* for 5 min. Then, the cell suspension from the centrifuged cell pellet was suspended in a T-75 flask with 1 mL of culture media.

#### 2.3.3. Cell Counting and Implantation

Quantifying cells was performed subsequent to the trypsinization, centrifugation, and suspension of the cell pellet in one milliliter of culture media. To accomplish this, in the 1.5 mL microtube, 10 µL of the homogenized cell suspension and 10 µL of trypan blue dye were each injected into 80 µL of growth media. Then, 10 µL of the solution was pipetted onto a hemocytometer slide. Under a microscope, the number of living cells in the four slide squares was counted, and the following method for calculating the quantity of cells per milliliter was used:

Number of cells/mL = (the sum of the cells of 4 squares/4) × the inverse of the dilution factor × 10^4^

#### 2.3.4. MTT Assay

The 3-(4,5-dimethylthiazol-2-yl)-2,5-diphenyltetrazolium bromide (MTT) was supplied in PBS at a 5 mg/mL concentration. In duplicates, roughly 5000 of each (L929 and 3T3) cell line were cultivated on the disks in each of the 96 wells, with the disks submerged in the culture medium. In order to establish individual disk toxicity, the plate was incubated at 37° C for 14 days in a humid atmosphere of a 5% CO_2_ and 95% O_2_ medium to conduct the test.

Each well received 100 µL of a 1×10 dilution of MTT solution (MTT starting stock: culture) and was incubated at 37 °C for three to four hours. The formation of formazan causes the medium’s color to turn purple. After removing the cell supernatant, 100 microliters of dimethyl sulfoxide (DMSO) were applied to each well to dissolve the formed crystals and incubated (15 min). Ultimately, the scaffolds were removed from the wells to read the plate.

#### 2.3.5. Analysis of Spectrophotometric Data and Cell Viability

The negative control samples all went purple, indicating that the cells were still alive; the higher the cell death rate, the lighter the color of the well. Finally, the spectrophotometer (Bio Tek ELx800, Bio Tek instruments, Winooski, VT, USA) measured the solution’s absorbance at a wavelength of 570 nm [30,31].

The formula below was used to compute the percentage of surviving cells [29,32,33]:

The survival (% of cell viability) percentage for each treatment sample = mean abs. of the treatment samples/mean abs. of negative control (distal water) × 100.

### 2.4. Degree of Conversion

Ten samples per group: CR, CR + 2% Chs, CR + 3% Chs, and CR + 5% Chs. Specimens’ dimensions were identical to those in prior tests. The degree of monomer conversion was obtained through the collection of Fourier transform infrared attenuated total reflection spectroscopy (FTIR-ATR) spectra using a spectrometer (Nicoler iS10, Omnic software, version 9.7.46, Thermo Fisher spectrometer, USA).

The abs. measurements were taken at a wavelength between 300 and 4000 cm^−1^ with a resolution of 4 cm^−1^ and 32 internal scans per reading. After the background calibration, the readings of Chs powder, uncured liquid resin, and other specimens’ spectra were recorded. Three spectra were obtained for each specimen, and the average was extracted.

**Figure 1:**
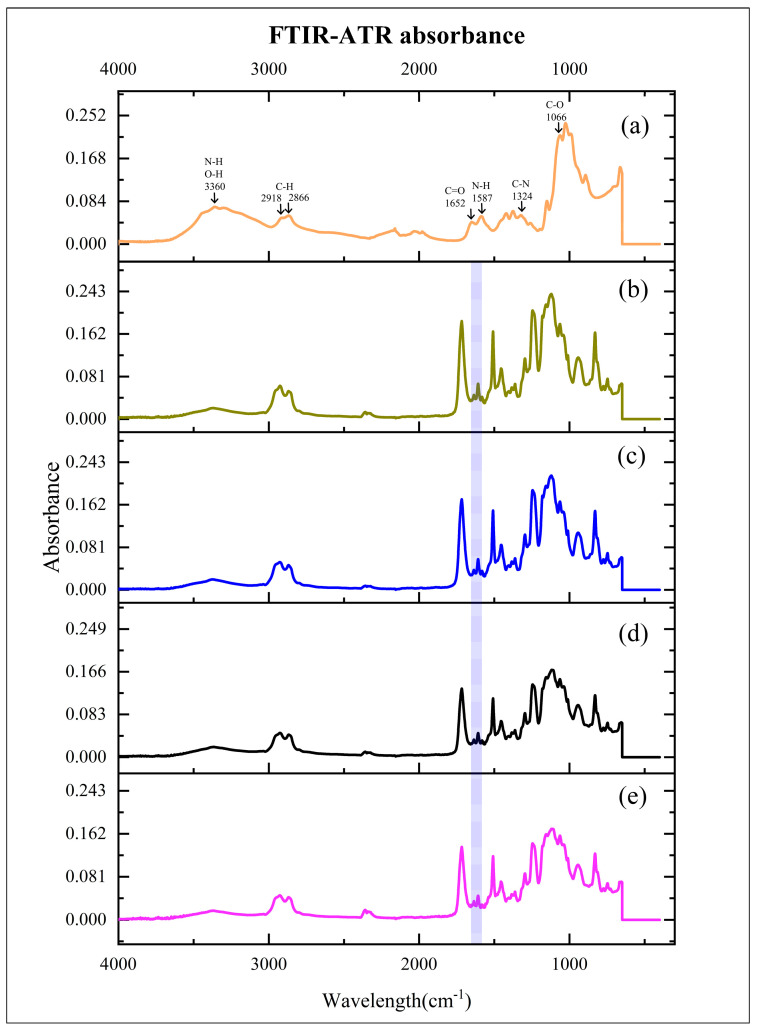
Mean absorbance values of experimented materials. (**a**): chitosan nanoparticles (Chs); (**b**): clear resin (CR); (**c**): 2% Chs incorporated into CR (CR + 2% Chs); (**d**): 3% Chs incorporated into CR (CR + 3% Chs); and (**e**): 5% Chs incorporated into CR (CR + 5% Chs). Highlighted area: absorbance values of the aliphatic C=C (1638 cm^−1^) and aromatic C=C (1608 cm^−1^) peaks.

Figure 1 reveals Chs powder, CR, and Chs incorporated into CR spectra. Each sample’s conversion degree was determined by calculating the abs. values of the aliphatic C=C (1638 cm^−1^) and aromatic C=C (1608 cm^−1^) abs. peaks using the following equation [34,35]:

Degree of conversion (%) = 1 − [(Abs. 1638 cm^−1^/Abs. 1608 cm^−1^) cured/ (Abs. 1638 cm^−1^/Abs. 1608 cm^−1^) uncured] × 100

### 2.5. Accuracy

Ten samples per group: Thr, CR, CR + 2% Chs, CR + 3% Chs, and CR + 5% Chs. For all samples, a maxillary typodont was used as a reference model to be surface scanned by a Primescan intraoral scanner to generate an STL of the reference scan. Concerning Thr aligners, a master model was first 3D printed via dental model resin (Model V3, Formlabs, Somerville, MA, USA). Second, the aligner was obtained using a thermoforming machine over the master model. The Thr aligners were processed following the manufacturer’s instructions, as mentioned above. On the contrary, an STL file of a passive aligner (1 mm in thickness) was designed for the reference scan using 3D in-house aligner software for manufacturing aligners (Bluesky Plan, version 4.9.4, Blue Sky Bio, Libertyville, IL, USA). Next, a clear aligner design was prepared for printing at 45° using the same 3D printing preparation software. 

After printing and post-printing processing (washing, curing, finishing, and polishing), each aligner was individually sprayed with a CAD/CAM spray (Diascan spray, Diaswiss, Nyon, Switzerland) to facilitate scanning, according to the manufacturer’s instructions. The aligners were then scanned using the same intraoral scanner mentioned above to generate a digital representation of each 3D-printed aligner’s intaglio surface. 

**Figure 2:**
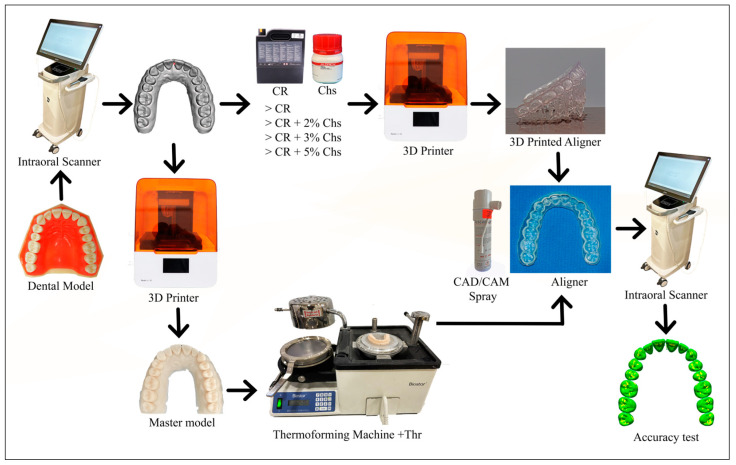
The accuracy test’s primary processes are depicted in the flow chart. The dental model was initially scanned to create a reference scan model (STL file). Then, the 3D printed aligners were created CR with varying concentrations of Chs. Meanwhile, the thermoforming aligners were created after the reference scan model was printed to obtain the master model, and later on, the thermoforming sheets were thermoformed over the master model (Thr). Following this, aligners were finalized and polished. Next, the internal surface of the aligners is scanned with the aid of a CAD/CAM spray to form an STL file for each aligner. They were then superimposed over the reference scan to determine the accuracy of the produced aligners.

The accuracy was evaluated via the pairwise superimposition of an STL file from a reference scan against the data from individual experimental groups (aligner’s scan file) [36], using an automated best-fit algorithm via the software (Geomagic Control X, version; 2022.1.0.70, 3D Systems, Rock Hill, SC, USA) [7,37]. The automatic best-fit alignment algorithm briefly applied a coarse alignment phase with a small number of comparison points between the surfaces of the two objects, followed by a fine adjustment phase with a larger number of comparison points to optimize the alignment. Each phase’s comparison points were determined automatically by the software. Any points in the scanned file that deviate in the positive or negative direction by more than the specified tolerance measurement were considered outside. Reports were generated for each superimposition, including average positive as well as negative standard deviations and percentage points outside the range [38]. Figure 2 reveals the sequence of steps in accuracy tests. 

The following equation was used by software to calculate the deviation between the reference scan and the aligner’s scan, and the data were calculated as the root mean square (RMS):

$$RMS=\frac{\sqrt{\sum_{i=N}^{N}{\left(x_{i}-{\hat{x}}_{i}\right)}^{2}}}{N}$$

where x is the point in the reference scan; $\hat{x}$ is the point in the aligner’s scan; i is the measurement; and *N* is the total number of points measured in each dataset. The color maps construed overall deviations to simplify intuitive contrasting, with deviations highlighted in a specific hue from –500 to +500 μm and values from –10 to +10 μm displayed in the same color.

### 2.6. Deflection Force

Ten samples per group (n = 10): Thr, CR, CR + 2% Chs, CR + 3% Chs and CR + 5% Chs. A 3-point bending test on a universal test machine (Cussons, Cussons Technology, Manchester, UK) was employed to determine its deflection force (length = 44 mm, width = 10 mm). The distance between the two machine base parts (8 mm) and the tip diameters was 1 mm [39]. The deflection was accomplished at a crosshead speed of 1 mm/min, and the stress was measured at 0.25 and 0.5 mm strain [40,41].

### 2.7. Tensile Strength

**Figure 3:**
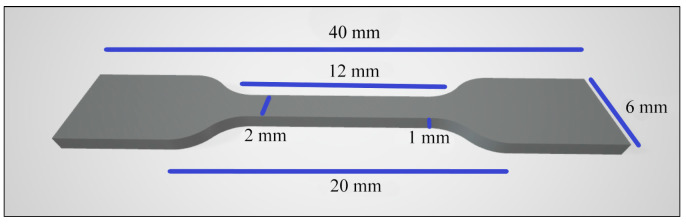
The tensile test specimen’s dimensions.

Ten samples per group (n = 10): Thr, CR, CR + 2% Chs, CR + 3% Chs, and CR + 5% Chs. The sample size was according to EN ISO 527-1:2012 [42,43], as shown in Figure 3. A universal strength testing machine (Cussons, UK) performed the tensile strength test. It measured megapascal (MPa), which required adaptor grips for tensile testing. The load was applied at a steady rate, in the range of 1 mm/min, and the force as well as deformation of the sample were measured continuously during the test at a temperature of 23 °C. The test continued until the sample fractured [39,42,43]. 

### 2.8. Statistical Analysis

The recorded data were analyzed using SPSS (version 23, IBM, USA). The anti-biofilm activity, cytotoxicity, degree of conversion, accuracy, deflection force, and tensile strength were tested and verified for the normality distribution of data via the Shapiro–Wilk test. A one-way analysis of variance (ANOVA) was then set at a significance level of *p* < 0.05, along with Tukey’s honestly significant difference (HSD) post hoc test for multiple comparisons. The null hypothesis is based on the fact that adding Chs has no substantial antibiofilm effect and does not modify the attributes, as mentioned earlier.

## 3. Results

### 3.1. Antibiofilm

**Figure 4:**
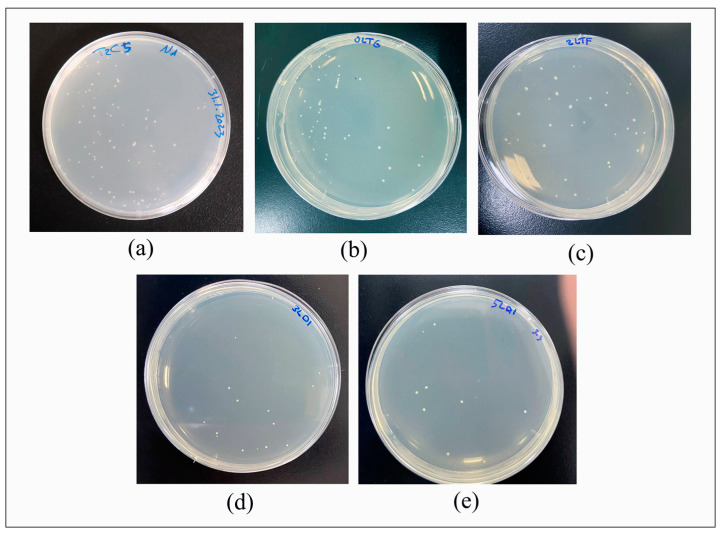
Direct culture technique for measuring colony-forming units per milliliter (CFU/mL). (**a**): Thr; (**b**): CR; (**c**): CR + 2% Chs; (**d**): CR + 3% Chs; and (**e**): CR + 5% Chs.

The number of CFU/mL of *Streptococcus mutans* determined via the direct cell culture technique is shown in Figure 4. Additionally, it revealed the CFU/mL values (×10^3^) of Thr: 48.15 ± 7.73; CR: 42.75 ± 12.17; CR + 2% Chs: 36.6 ± 7.88; CR + 3% Chs: 19.3 ± 8.84; and CR + 5% Chs: 11.7 ± 6.16.

**Figure 5:**
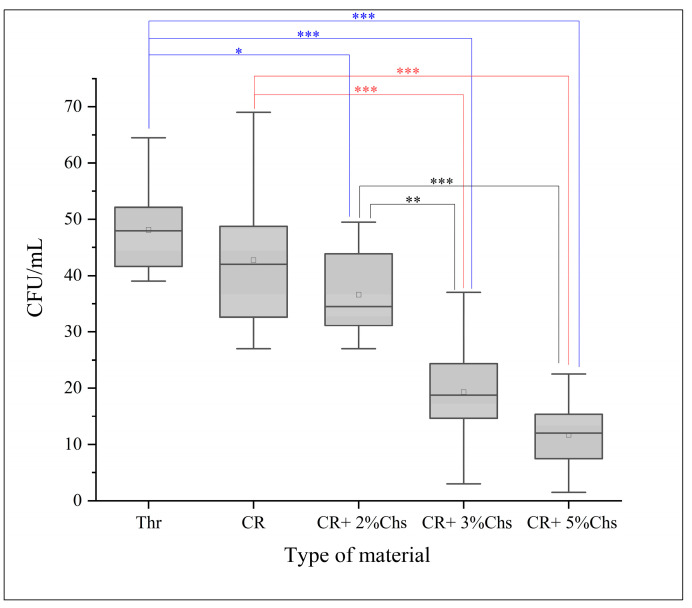
Representative means of CFU/mL (10^3^) of the experimental groups. The post hoc test (Tukey’s HSD) was applied to reveal the group differences. Note: *: *p* < 0.05; **: *p* < 0.01; ***: *p* < 0.001; and error bar: standard deviation = 1.

Figure 5 illustrates the impact of Chs on the reduction in mean CFU/mL, which is highly significant, as the outcome of the ANOVA test indicated statistically significant differences in the CFU/mL values of the various groups (*p* < 0.001). Tukey’s HSD post hoc test for multiple comparisons showed statistically significant differences in the CFU/mL values of CR + 3% Chs and CR + 5% Chs materials when compared to other groups, and there were no significant differences between Thr and CR, CR and CR + 2% Chs, and CR + 3% Chs and CR + 5% Chs (*p* > 0.05).

### 3.2. Cytotoxicity

The descriptives of cell viability are presented in Table 2. The analytical test for the mean percentage of cell viability (cytotoxicity) presented a non-significant difference between the tested groups (Thr, CR + 2% Chs, CR + 3% Chs, and CR + 5% Chs) (*p* > 0.05). 

### 3.3. Degree of Conversion

**Figure 6:**
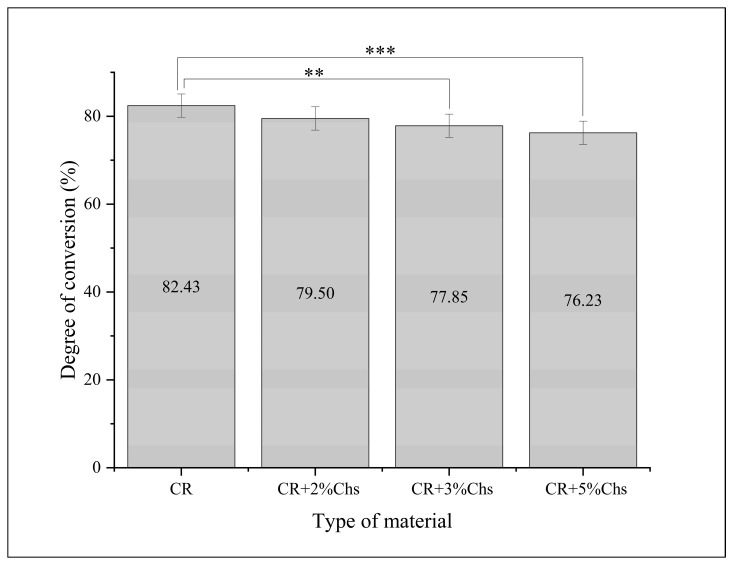
Means and differences in the percentage of the degree of conversion between different groups. The post hoc test analyzed the group differences. Note: **: *p* < 0.01; ***: *p* < 0.001; and error bar: standard deviation = 1.

The descriptive values and differences are displayed in the bar graph (Figure 6). The inferential statistic (ANOVA) test resulted in a significant difference between the experimental materials regarding the degree of conversion generated from the difference between CR and CR + 3% Chs (6%) as well as CR and CR + 5% Chs (8%) (Tukey HSD), whereas the difference was not significant between other groups.

### 3.4. Accuracy 

**Figure 7:**
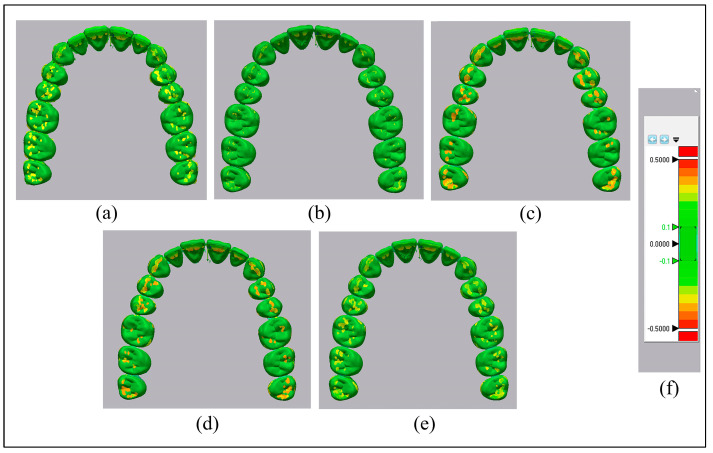
The color mapping highlights the accuracy of aligners. (**a**): Thr; (**b**): CR; (**c**): CR + 2% Chs; (**d**): CR + 3% Chs; (**e**): CR + 5% Chs; and (**f**): color grading map.

The mean values of discrepancies in the Thr group were significantly higher compared to those of the other groups, as exhibited in the color map displayed in hues (Figure 7). The RMS reveals the accuracy of different groups, which is illustrated in Table 3. The ANOVA test was performed, and it showed a highly significant increase in the RMS in the Thr group (less accuracy) compared to all of the other groups (*p* < 0.001). In contrast, no significant difference between the other groups was found.

### 3.5. Deflection Force

According to the results, the mean deflection force (MPa) at (0.25, 0.5) mm increased with an increase in the concentration of Chs, as shown in Table 4. The analysis (ANOVA) showed significant differences between the groups at strains of 0.25 mm and 0.5 mm. At a strain of 0.25 mm, these resulted from the differences between CR and CR + 5% Chs (*p* < 0.05), Thr and CR + 5% Chs (*p* < 0.001), Thr and CR + 2% Chs (*p* < 0.01), and CR + 3% Chs and CR + 5% Chs (*p* < 0.05). At a strain of 0.5 mm, significant differences were found between CR and CR + 5% Chs and Thr and CR + 2% Chs (*p* < 0.01), as well as Thr and CR + 3% Chs and CR + 3% Chs and CR + 5% Chs (*p* < 0.05); a highly significant difference was found between Thr and CR + 5% Chs (*p* < 0.001). 

### 3.6. Tensile Strength

The mean values of the tensile strength (Thr: 47.54 ± 3.83; CR: 63.71 ± 1.99; CR + 2% Chs: 54.10 ± 2.36; CR + 3% Chs: 53.62 ± 3.17; and CR + 5% Chs: 48.16 ± 3.15) in MPa are depicted in Table 5. The bar chart demonstrates the mean values and the post hoc test outcomes for multiple comparisons. The ANOVA disclosed highly significant differences between the categories CR and CR + 2% Chs, CR and CR + 3% Chs, CR and CR + 5% Chs, CR and Thr, CR + 2% Chs and CR + 5% Chs, CR + 2% Chs and Thr, and CR + 3% Chs and Thr (*p* < 0.001), and a significant difference (*p* < 0.01) between CR + 3% Chs and CR + 5% Chs. On the contrary, non-significant differences were found between CR + 2% Chs and CR + 3% Chs, and Thr and CR + 5% Chs (*p* > 0.05).

## 4. Discussion

### 4.1. Antibiofilm

Besides other manufacturer recommendations for various appliances, the resin (Dental LT clear resin) is frequently employed in research as a mechanically, physically, and biologically promising 3D printed resin for directly produced aligners [5,24,38,44,45]. In the current study, Chs have been incorporated into the CR. With the addition of Chs, an FTIR-ATR examination confirmed the presence of alterations in the polymer’s spectra. The antibiofilm advantage of Chs as an active component of investigated material depends on many factors: medium, pH, pathogen type, structural features, the proportion of acetylation, molecular weight, concentration, and the nanomaterial source [46,47]. The produced Chs utilized in the current study has a degree of deacetylation of 83.5%, an average particle size of 74 nm [48], and a molecular weight of 50–190 kDa, with antibiofilm activity, as shown in a study [49].

Antibiofilm activity was manifested by reducing viable bacterial adhesion to the surface of the newly produced material, as compared to the resin alone or Th materials. The results confirmed that anti-adhesion activity after adding 3% and 5% Chs to the resin led to a significant decrease in colony-forming units by 31% and 70%, respectively, compared to Thr. 

The efficacy in preventing biofilm formation is measured, which is the primary cause of dental caries and periodontal disease. The mechanism-specific assessment of antibiofilm efficacy emphasizes evaluating bacterial growth suppression or death and preventing initial bacterial adhesion [50,51]. Antibiofilm activity can be attributed to Chs’ mechanism of action, which involves disrupting the cell wall, interacting with the membranes, disrupting transmembrane and electron transport, organelle disruption and leakage, and inhibiting deoxyribonucleic acid (DNA) replication, which results in toxin production and microbial growth inhibition, which subsequently result in cell damage and death [52,53].

### 4.2. Cytotoxicity Assay

The cytotoxicity profile of Chs is dependent on numerous elements, such as the qualities of the parent material used to synthesize the nanoparticles, the size of the nanomaterial, the interacting cell types, and the degree of deacetylation; the latter has a bigger influence than molecular weight on the absorption and cytotoxicity of Chs [54].

The aligner material’s cytotoxicity is influenced by temperature, humidity, oxygen exposure, light exposure, the post-rinsing procedure, material molding, sterilization, the ratio of sample surface area versus cell layer or culture medium, and extracts [29,55]. The aligner materials were incubated in the cell culture media for two weeks and categorized according to the duration of tissue contact as “prolonged exposure devices,” which refer to devices that contact the tissue for 1–30 days [56]. The greater the viability percentage, the lower a test item’s cytotoxic potential. The results demonstrated a low level of cytotoxic potential of the CR, and after adding Chs, they also had a comparable percentage to Thr aligner material. The cytotoxic potential against cell lines is graded using the ISO 10993 categorization system by the percentage of cell viability, which states that over 90% cell viability is considered to have no cytotoxic effect. Cell viability between 60% and 90% was regarded as minimal cytotoxicity. This result coincides with other studies that tested the biocompatibility of the aligner material [10,55,57].

### 4.3. Degree of Conversion

The resin’s FTIR-ATR spectra analysis measured the degree of conversion of the methacrylate double bond, as it was noted that the remaining unpolymerized polymer could be determined much simpler and more reliably by directly comparing the intensity of the bands at 1638 cm^−1^ [58]. 

The findings showed a slight decrease in the proportion of the degree of conversion. The evidence revealed that this change does not significantly sway the level of polymerization, which might be due to the crosslinking behavior of the Chs [59]. Another possible contributing factor is the blade movement in the resin tank during the 3D printing of each layer, which mixes the materials and leads to better homogenization of the fillers in the resin. Additionally, the heating processes before and during polymerization are also considered to affect the level of polymerization [60]. Additionally, the intensity of the curing light projection, post-polymerization protocol, temperature, time, and humidity might affect the conversion level and overall material quality [35,61,62].

### 4.4. Accuracy

The accuracy of 3D images and ultimately produced aligners is multifactorial and influenced by the scanner type, operator skill, scanning field and technique [63], room lighting conditions [64], and the application of CAD/CAM spray [65]. The digitized offset might also modify surface reproducibility, defined as a non-customizable internal gap built by the software that is integrated to facilitate the sitting of an aligner [66].

Additionally, the resin type, 3D printer deployed, layer thickness during 3D printing, and printing angulations also implicate the accuracy and qualities of 3D printed resin materials [67,68,69]. Finally, 3D-printed post-processing and storage are also claimed to modify reproducibility [70]. 

The accuracy of produced 3D printed aligners was superior to Th (CR = 13.5%, CR + 2% Chs = 12.6%, CR + 3% Chs = 12%, and CR + 5% Chs = 9.9%), in harmony with prior research findings in the realm of 3D printed and thermoformed aligners [7,66,70]. The inclusion of Chs did not hamper the precision of 3D printing, and the differences (less than 300 µm) exhibited by various variables are deemed clinically acceptable [71].

### 4.5. Deflection Force

This study tested deflection because it evaluates a material’s resistance to bending or twisting and the scale of force generated by an aligner. According to the literature, the deflection force is affected by the degree of mismatch or activation, the material properties of the aligner, the force’s direction, which is determined by the orientation of the active surface (adjacent to that surface), and variations in intraoral temperature [21,72,73,74]. The examined material’s viscoelastic properties prevented it from breaking during a three-point bending test. According to the data, the deflection force at strain levels of 0.25 and 0.5 mm exhibited an indiscernible effect of Chs percentage, specifically when it reached 3%. However, the findings demonstrated that increasing the Chs (CR + 5% Chs) percentage in the CR led to approximately 13% more force (MPa) generation at both deflection values.

### 4.6. Tensile Strength

The data delineated that the presence of Chs decreased the tensile strength of the resin, nevertheless within an acceptable limit compared to Thr, and complied with other studies’ findings considering the extent of filler dispersion, size, and aspect ratio [75,76,77]. Moreover, this might be due to reinforcement and the enhanced interfacial bonding effect of Chs dispersed in resin due to their high surface area, producing a network-like structure. This network strengthens the composite material and raises the stress distribution and modulus of elasticity [59,78]. Meanwhile, Chs can restrict polymer chain mobility in the resin matrix, increasing the stiffness and modulus of elasticity [79].

To mitigate the influence of material thickness and post-processing procedures on the mechanical characteristics of the 3D-printed specimens, a standard thickness of 1 mm was applied to the samples. Additionally, the same operator uniformly polished the samples, following a prescribed procedure [72,80]. 

The limitations of this study were that the experiment was performed in vitro. The antibacterial test antibiofilm only measured colony formation by one strain of bacteria. Additionally, the degree of color variation might be significant at a high mixture level.

Suggestions: testing the material in vivo in humans and adding other nanoparticles in different concentrations might affect the stated points. Furthermore, cycling might also affect material properties.

## 5. Conclusions

With the limitations of this study, the enhancement of the antibiofilm effect of 3D printed aligners against one of the main cariogenic bacteria (*Streptococcus mutans*) by adding Chs at certain concentrations without compromising the CR’s cytotoxicity, degree of conversion, accuracy, and within a clinically acceptable range of deflection force and tensile strength, might be a promising strategy to help prevent or eliminate dental caries by reducing biofilm formation associated with orthodontic therapy. These findings suggest that Chs are a step toward more competent dental treatments to improve the properties of aligner treatment.

## Figures and Tables

**Table 1:** Demonstration of the clear resin (CR) composition applied in this study.

Ingredients	Percentage
Methacrylic oligomer	>70
Glycol methacrylate	<20
Pentamethyl-piperidinyl sebacate	<5
Phosphine oxide	<2.5

**Table 2:** The effect of CR, CR + 2% Chs, CR + 3% Chs, CR + 5% Chs, distal water (DW), polyethylene (PE), and ethanol (ET) on the mean percentage of cell viability (L929 and 3T3) cell lines.

Cell Viability	Type of Material	Mean	Std. Deviation	Std. Error	95% Confidence Interval for Mean		Min.	Max.
					Lower Bound	Upper Bound		
L929	DW	100	7.81	3.49	90.3	109.7	92.18	111.69
	Polyethylene PE	88.12	18.05	8.07	65.71	110.53	70.72	111.42
	Thr	101.8	14.11	6.31	84.27	119.32	87.82	120.41
	CR	93.86	9.86	4.41	81.61	106.10	82.65	107.77
	CR + 2% Chs	110.81	15.7	7.02	91.32	130.3	92.18	131.99
	CR + 3% Chs	95.01	12.1	5.41	79.99	110.03	82.38	111.86
	CR + 5% Chs	108.26	11.04	4.94	94.55	121.97	98.47	123.44
	ET	30.55	5.30	2.37	23.97	37.13	25.29	37.94
3T3	DW	100	11.71	5.24	85.46	114.54	81.87	112.8
	PE	91.85	6.4	2.86	83.91	99.8	82.07	97.8
	Thr	92.22	7.09	3.17	83.42	101.02	84.22	100.45
	CR	96.47	4.9	2.19	90.38	102.56	89.42	101.57
	CR + 2% Chs	85.81	8.87	3.97	74.8	96.83	78.09	100.35
	CR + 3% Chs	90.67	8.19	3.66	80.5	100.84	78.4	100.55
	CR + 5% Chs	90.24	7.84	3.51	80.5	99.98	81.16	101.37
	ET	36.87	6.05	2.71	29.36	44.39	28.38	42.47

**Table 3:** Root mean square (RMS) in µm of the deviation values for measuring the accuracy.

Type of Material	Mean	Std. Deviation	Std. Error	95% Confidence Interval for Mean		Min.	Max.
				Lower Bound	Upper Bound		
Thr	272.23	20.3	6.42	257.72	286.75	236.8	293.81
CR	235.5	1.96	0.62	234.1	236.9	233.08	239.34
CR + 2% Chs	237.84	8.02	2.54	232.1	243.57	223.36	251.72
CR + 3% Chs	239.56	2.04	0.65	238.1	241.01	235.93	241.96
CR + 5% Chs	245.22	1.55	0.49	244.1	246.33	243.57	248.54

**Table 4:** Descriptive values of deflection force (MPa).

Deflection	Type of Material	Mean	Std. Deviation	Std. Error	95% Confidence Interval for Mean		Min.	Max.
					Lower Bound	Upper Bound		
0.25 mm strain	Thr	8.96	0.53	0.22	8.4	9.52	8.28	9.6
	CR	10.04	0.82	0.37	9.18	10.9	9.12	11.28
	CR + 2% Chs	10.80	0.79	0.32	9.98	11.62	9.6	11.64
	CR + 3% Chs	10.06	0.74	0.3	9.29	10.83	9.12	10.92
	CR + 5% Chs	11.38	0.58	0.24	10.77	11.99	10.44	12.00
0.5 mm strain	Thr	12.38	0.62	0.25	11.73	13.03	11.64	13.32
	CR	14.51	2.05	0.84	12.36	16.67	12	17.04
	CR + 2% Chs	16.37	1.42	0.58	14.87	17.86	13.8	17.52
	CR + 3% Chs	15.56	2.68	1.094	12.75	18.37	12.84	18.72
	CR + 5% Chs	18.72	1.39	0.57	17.26	20.18	16.32	20.4

**Table 5:** The descriptive values demonstrate the tensile strength (MPa).

Type of Material	Mean	Std. Deviation	Std. Error	95% Confidence Interval for Mean		Min.	Max.
				Lower Bound	Upper Bound		
Thr	47.54	3.83	1.21	44.8	50.28	42.95	55.65
CR	63.71	1.99	0.63	62.28	65.14	61.05	66.75
CR + 2% Chs	54.1	2.36	0.75	52.41	55.79	50.75	58.95
CR + 3% Chs	53.62	3.17	1	51.35	55.89	49.25	57.55
CR + 5% Chs	48.16	3.15	1	45.91	50.41	42.6	52.1

## Data Availability

The data that validate the findings of this investigation are available upon reasonable request from the corresponding author.
